# Cancer immune exclusion: breaking the barricade for a successful immunotherapy

**DOI:** 10.3389/fonc.2023.1135456

**Published:** 2023-05-22

**Authors:** Sofia Bruni, María Florencia Mercogliano, Florencia Luciana Mauro, Rosalia Inés Cordo Russo, Roxana Schillaci

**Affiliations:** Laboratorio de Mecanismos Moleculares de Carcinogénesis. Instituto de Biología y Medicina Experimental (IBYME-CONICET), Buenos Aires, Argentina

**Keywords:** tumor microenvironment, immune exclusion, physical barrier, myeloid cells, extracellular matrix, tumor infiltrating lymphocytes (TILs), tumor-associated vasculature, tumor-associated macrophage (TAMs)

## Abstract

Immunotherapy has changed the course of cancer treatment. The initial steps were made through tumor-specific antibodies that guided the setup of an antitumor immune response. A new and successful generation of antibodies are designed to target immune checkpoint molecules aimed to reinvigorate the antitumor immune response. The cellular counterpart is the adoptive cell therapy, where specific immune cells are expanded or engineered to target cancer cells. In all cases, the key for achieving positive clinical resolutions rests upon the access of immune cells to the tumor. In this review, we focus on how the tumor microenvironment architecture, including stromal cells, immunosuppressive cells and extracellular matrix, protects tumor cells from an immune attack leading to immunotherapy resistance, and on the available strategies to tackle immune evasion.

## 1 Introduction

The tumor microenvironment (TME) is an heterogenous milieu composed not only by cancer cells, but also by different cell types that can contribute to tumoral escape of immune surveillance and dampening of responses to immunotherapy. These cells include myeloid-derived suppressor cells (MDSCs), tumor-associated macrophages (TAMs), tumor infiltrating lymphocytes (TILs), cancer associated fibroblasts (CAFs), blood vessels and the extracellular matrix (ECM), composed of collagen and proteoglycans (1, 2).

Immunotherapy is a field that has been rapidly developing over the past few decades, and immune checkpoint blockade (ICB) has demonstrated a tremendous contribution towards novel therapeutic targets and effective drugs that are now used as standard therapy for several cancers, which show in most cases durable responses and extensions of survival curves. However, it is known that responses are patient-dependent and resistance events hamper their clinical benefit. One of the resistance mechanisms proposed for ICB is the inability of lymphocytes to infiltrate the tumor, also known as immune exclusion. This phenomenon can be due to activated oncogenic pathways (3), hypoxia (4–8), degenerated blood vessels (9, 10), cytokines and chemokines released by tumor (11), stromal cells, immune infiltration of immunosuppressive cells (12) and ECM that limit lymphocyte access to the tumor nest. In this review, we summarize the different mechanisms by which physical barriers may promote immune exclusion and potential therapies to overcome it.

## 2 Immune exclusion

With the advent of ICB and adoptive cell therapy (ACT), treatment of solid and hematologic malignancies has been revolutionized. However, not all patients benefit from these therapies. It is imperative to elucidate which are the mechanisms of immune resistance that limit the efficacy of immunotherapy (13). A predominant phenomenon observed in cancers that do not respond to immunotherapy is the absence of dialogue between the immune cells and the tumor (14). Immune exclusion is a complex phenomenon in which T cells are recruited to the tumor periphery by chemoattraction and antigenic stimuli that promote their persistence, but existing barriers prevent T cells from infiltrating tumor nests and killing cancer cells. The presence of TILs on the tumor core has been proved to have prognostic value in different cancer types (15–21). Therefore, the impairment of these immune cells in reaching the tumor nests and mounting an efficient immune response against the tumor is an issue of clinical relevance.

### 2.1 Tumor immunophenotypes

Clinical studies have defined immune profiles that can predict responses to immunotherapy. According to this, three basic immune profiles can be identified: immune inflamed, immune excluded and immune desert phenotypes (14). The immune inflamed tumors are characterized by the presence of CD4+ and CD8+ T cells and myeloid cells in the tumor parenchyma. Immune cells are close to, or in contact with tumor cells. For this reason, they usually respond to ICB therapies. However, not all patients respond, indicating that immune cell infiltration is not sufficient to induce an effective immune response probably caused by T cell exhaustion (22). Therapies with ICB have shown promise in melanoma (23), non-small-cell lung cancer (NSCLC) (22, 24) and urothelial cancer (25, 26), which are examples of the inflamed subtype. The immune-excluded tumors present no infiltration of CD8+ T cells to the tumor core, but have accumulation of them around tumor margins, and are resistant to multiple types of treatment (27). This immunophenotype encompasses the most harmful malignancies, including pancreatic ductal adenocarcinoma (PDAC) (28, 29), breast (30–33) and ovarian cancer (34). The immune desert tumors are characterized by a lack of T cells in the tumor stroma (35–38). Although myeloid cells may be present, few or no CD8+ T cells are present resulting in a non-inflamed TME. As expected, such tumors rarely respond to ICB therapies (37). This immunophenotype likely reflects the absence of pre-existing antitumor immunity, suggesting that tumor-specific T cell generation is the rate-limiting step (14). An example of this immunophenotype is glioblastoma, which is further characterized by a lack of tumor antigens, defects in antigen presentation, and a high accumulation of immunosuppressive cells (39). Both the immune desert and the immune excluded phenotypes can be considered non-inflammatory tumors. ICB therapies have shown promise in immune-inflamed tumors, however, such success is not extended to immune-excluded or desert tumors (40, 41). Therefore, therapies promoting the conversion of these latter two immunophenotypes into inflamed tumors could help reduce resistance to ICBs and provide a new treatment option (27, 42).

### 2.2 Clinical relevance of TILs

A study carried out by Kather et al. in 177 samples of patients with cancers of different histology, evaluated the topography of immune infiltration using immunohistochemistry. Samples were classified according to the number of cells per mm (2) into three spatial compartments: outer invasive margin (0–500 μm outside the tumor invasion front), inner invasive margin (0–500 μm inside the tumor invasion front), and in the tumor core (>500 μm inside the invasion front). It was found that there was a correlation between the infiltration of the tumor core and the internal invasive margin (43). In this way, different types of tumors were grouped into 3 topographies: high density outside of the tumor with a low density inside the tumor, which can be described as “immune excluded”, low density inside and outside represents “cold” tumors, and high density inside the tumor is classified as “hot” regardless of cell density outside the tumor. A similar approach was carried out by Galon et al. They observed that the infiltration of CD8+ T cells within tumor nests combined with their peri-tumoral presence predicts improved survival of patients with colorectal cancer (CRC) with higher accuracy than the classical TNM staging (44). Moreover, limited observations in head and neck squamous cell carcinoma suggests that immune-excluded cancers are transcriptionally indistinguishable from the immune inflamed ones. Therefore, the distinction between these phenotypes is mainly due to the spatial resolution and localization of the immune cell populations (43, 45).

In large cohorts of early triple-negative breast cancer (TNBC) patients, mostly treated with standard adjuvant therapy, a higher total CD8+ and CD4+ TILs count is significantly associated with favorable outcomes (46–48). However, carrying out a more exhaustive analysis and sub-classifying tumors with different patterns of immune infiltration could be of help when evaluating prognosis. A recent report identified four distinct subtypes of tumors based on their surrounding TME, immunodesert (ID); restricted margin (MR); restricted stroma (RS) and fully inflamed (FI), by integrating gene expression signatures with spatial patterns of CD8+ T cell localization in intratumoral and stromal matched samples of treatment-naive TNBC. Both MR and ID tumors were characterized by low CD8+ infiltration (with the first showing CD8+ accumulation at the margins) and these patients were associated with a poorer disease prognosis. In contrast, FI and SR tumors were characterized by abundance of CD8+ T cells, with SR tumors showing stromal-restricted CD8+ infiltration, and FI showing CD8+ T cell infiltration within the stromal and epithelial compartments. Both FI and SR patients were associated with a more favorable prognosis (49).

While in the previously cited works the authors have focused mainly on making topographic distinctions to classify tumors with different patterns of immune infiltrate, other authors have opted to use gene expression profiles as a prognostic marker that could potentially be included in the clinical practice. ICB effectiveness can be affected by the degree of CD8+ T cell infiltration (50), mutation or neo-antigen load (23), PD-L1 level (51), antigen presentation defects (52), interferon signaling (53), mismatch repair defIciency (54), tumor aneuploidy (55) and intestinal microbiota (56). However, none of these factors are enough to achieve accurate outcome predictions (51). Predicting tumor response to ICB requires an understanding of how tumors escape the immune system. There are two distinct mechanisms of tumor immune escape (38, 57). Some tumors have a high level of CD8+ T cell infiltration, but these cells are dysfunctional. In other tumors, immunosuppressive factors may exclude T cells from infiltrating tumors (58). Jiang et al. developed a computational framework, called Tumor Immune Dysfunction and Exclusion (TIDE), to identify factors that underlie these two mechanisms of tumor immune escape. TIDE integrated and modeled data from 189 human cancer studies and validated an accurate gene signature to model tumor immune escape that could serve as a reliable surrogate biomarker to predict ICB response. TIDE predicted the outcome of melanoma patients treated with first line anti-PD1 or anti-CTLA-4 more accurately than other biomarkers, such as PD-L1 level and tumor mutation burden (59). Finally, other authors have resorted to computational tools to study the TME. In recent years, computational imaging approaches originating from artificial intelligence have achieved success in automatically quantifying radiographic characteristics of tumors (60–62). Radiomics is an emerging field within medical research that aims to use advanced imaging analysis to study tumors and potentially predict treatment outcomes based on radiological features. Jazieh et al. demonstrated that a radiographic image-based biomarker on baseline CT scans is significantly associated with progression-free survival and overall survival (specifically prognostic within individual PD-L1 categories) in patients with NSCLC treated with durvalumab (anti PD-L1 monoclonal antibody) after chemoradiotherapy (CRT) or CRT alone by using radiomic texture patterns within and outside the NSCLC tumor cells (63).

Clinical distinction between the above-mentioned tumor immunophenotypes is highly important for the design of next-generation immunotherapy agents and the possibility of a tailored medicine (64). To determine the prevalent mechanisms of immune exclusion and address them to expand the effectiveness of immunotherapy approaches must be of first priority for researchers and physicians. Among those mechanisms, we can mention physical barriers that will be discussed in the next section of this review, and also metabolic and functional barriers (65) that prevent T cell infiltration to the tumor core and cancer cells elimination (45).

## 3 Physical barriers within the TME

### 3.1 Extracellular matrix

It is well known that the ECM plays a crucial role in the interaction of cells within a tissue (66) and, for this reason, it is of high importance during tumor development and progression (67). Interactions between the ECM and the tumor go in both directions: the ECM determines the morphology of the cells, their differentiation and migration, and regulates cell-cell interactions. On the other hand, cells actively remodel the composition and geometry of the ECM in favor of tumor progression (68–71). ECM dysregulation during cancer progression is mainly carried out by stromal cells, including CAFs and immune cells (72, 73). Nevertheless, epithelial cells and mesenchymal stem cells may also be involved at late stages of cancer development (74, 75). How the ECM changes along tumor establishment and progression is well reviewed elsewhere (67, 76). The ECM also has immunostimulants and, hence, potential antitumoral effects. In this sense, it has been reported that ECM components such as biglycan, heparan sulfate or versican can modulate leukocyte (77) and dendritic cells recruitment (78), have an active role in inflammation (79), T cell activation and macrophage recruitment (80), among others. In this review we will focus on the pro-tumoral effects of different components of the ECM and their impact on the efficacy of immunotherapies, since the immunostimulant effects have been reviewed elsewhere (81–84).

**Figure 1:**
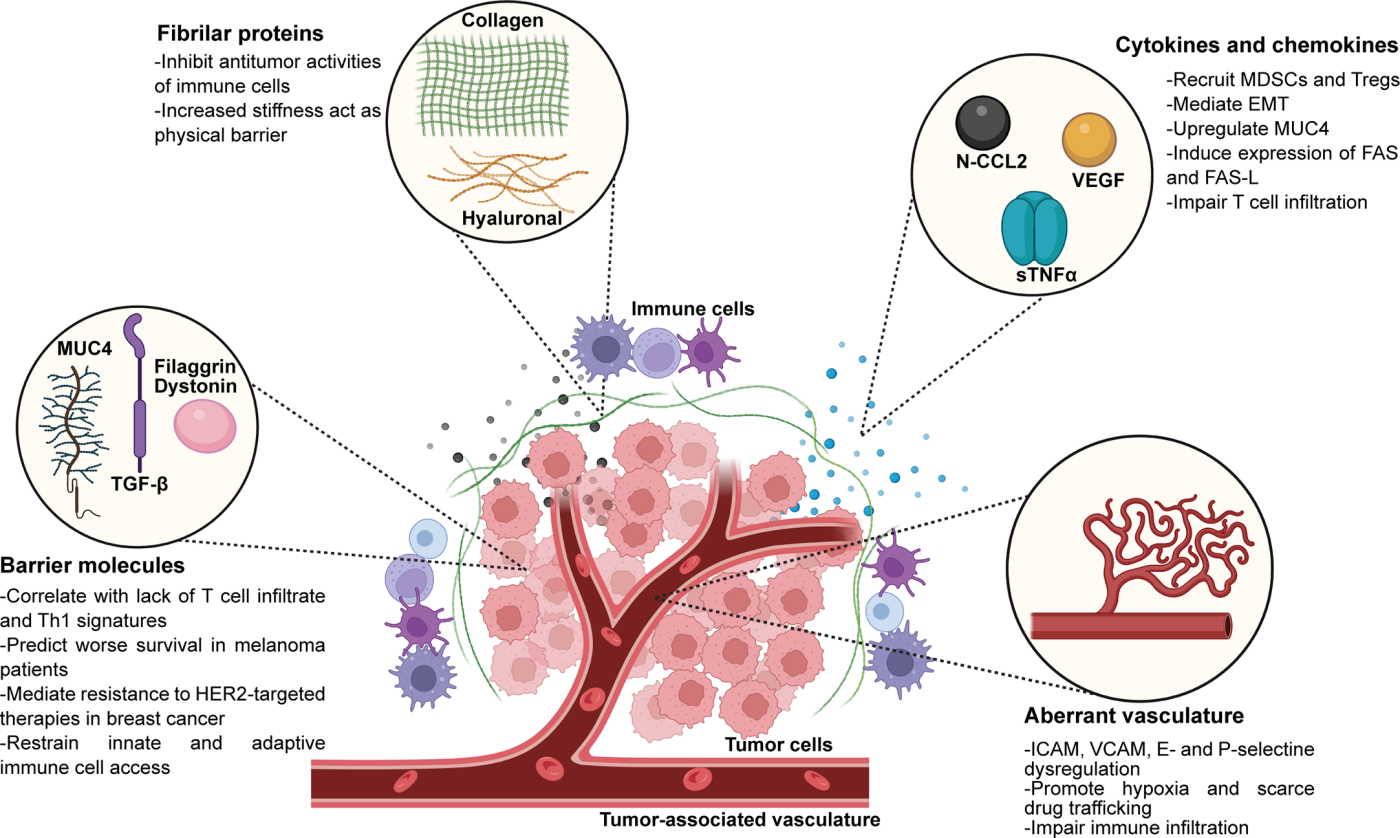
Extracellular matrix components present in the TME that play active roles in immune exclusion and immunosuppression. EMT, epithelial-mesenchimal transition. Created with BioRender.com.

The components of the ECM can be divided into two categories: the liquid part, that include cytokines and chemokines, and the polymeric scaffold. The latter contains different types of polymers such as polysaccharides, like hyaluronan and chondroitin sulfate, biglycan and perlecan, and fibrous proteins, like collagen and fibronectin that provide structure to the matrix and participate in cell-cell interactions (67). The ECM plays a key role in the establishment of anti-tumor immune escape mechanisms since it allows the diffusion of signaling (such as cytokines and chemokines), the recruitment of immune cells into the tumors, as well as the interaction between specific ligands and receptors. There are large body of evidence that have described how the ECM components contribute to immune exclusion, like the contribution of chemokines and cytokines, barrier molecules, collagen and hyaluronic acid and the tumor-associated vasculature, that is discussed in the section below and summarized in **Figure 1**.

#### 3.1.1 Chemokines, cytokines and barrier molecules

Chemokines released from cells immersed in the TME account for recruitment and extravasation of different immune cell populations. However, the TME can modify chemokines posttranslationally to allow differential cell infiltration and to promote immune exclusion. One example is the production of reactive nitrogen species by MDSCs, which induces nitration of chemokine (C-C motif) ligand 2 (N-CCL2) (85). CCL2 is secreted by different cell types, like T cells, monocytes, and also tumor cells, and works as a chemoattractant for myeloid cells, activated CD4+ and CD8+ T cells, and NK cells (86–89). Moreover, CCL2 has the ability to trigger granule release from both NK and CD8+ T cells (90). The authors prove that nitration of this cytokine results in T cells being trapped within the stroma surrounding human prostate and colon cancer cells (85). In contrast, N-CCL2 attracts MDSCs and TAMs to the tumor core tissue, potentially contributing to the differential recruitment to the TME of these immunosuppressive immune cell types. Interestingly, drugs that inhibit nitration of CCL2 proved to enhance TILs accumulation in preclinical models, and resulted in improved efficacy of adoptive cell therapy. Another important contributor to the composition and abundance of the ECM is the cytokine transforming growth factor-β (TGF-β). This molecule is secreted mainly by CAFs, but it can also be produced by myeloid cells and tumor cells (91–94). TGF-β has several key functions as a major regulator of the ECM homeostasis. For example, it stimulates CAFs to produce structural collagen fibers. Also, this cytokine induces the synthesis of enzymes involved in degradation or crosslinking of collagens (95), thus regulating fibrosis and stiffening of the ECM. Collagen not only acts as a physical barrier to T cell infiltration, but it can also act as a ligand for a vast number of receptors present in tumor and immune cells. One example is the leukocyte-associated immunoglobulin-like receptor-1 (LAIR-1), an immune checkpoint that is expressed on the surface of immune cells. When collagen XVII fibers present in the ECM surrounding the tumor binds to LAIR-1, the signal transduction results in inhibitory signals that cause T cell exhaustion and impairment of NK cell, monocyte and dendritic cell activation and proper function (96). Recently, it has been proved that T cell exclusion and poor response to ICB therapies is associated with a gene signature of TGF-β activation (97–99). Particularly in metastatic urothelial immune-excluded tumors that show no response to atezolizumab, the authors found that there was an enrichment of a fibroblast TGF-β gene signature, and that the expression of this gene signature was directly linked to T cell trapping in the collagen-rich stroma surrounding the tumor (97). Moreover, they demonstrated that the co-inhibition of TGF-β and PD-L1 transformed tumors from excluded to inflamed, adding to the idea that TGF-β signaling restricts T cell infiltration to the TME and hampers efficient antitumor immune responses. Also, TGF-β secreted by stromal cells is able to induce the expression of the vascular endothelial growth factor (VEGF) to promote tumor angiogenesis, and recruit immunosuppressive cells like MDSCs and Tregs, to further enhance immune escape and impair T cell infiltration. Furthermore, Horn and collaborators demonstrated that in two collagen-rich murine carcinomas, MC38 colon and EMT6 breast, the inhibition of LAIR-1 and TGF-β plus anti-PD-L1 therapy was able to control tumor growth and reshape the collagen-rich ECM (100). Authors evidenced that the combined treatment promoted an increased tumor infiltration and activation of CD8+ T cells, and was also able to repolarize protumoral macrophages to the antitumoral subtype. Moreover, they proved that this treatment resulted in high tumor regression rates and long-term protection by tumor-specific immune cells (100). TGF-β also mediates the epithelial-to-mesenchymal transition (EMT) (101, 102), which has been described as another process that raises mechanical barriers for T cell infiltration in various cancers, such as melanoma, ovarian and gastric (103–106). Another cytokine that plays an important role in cancer progression and immune exclusion is the pleiotropic cytokine TNFα. We have previously reviewed the participation of TNFα on the progression and metastasis of different breast cancer subtypes and the antitumor immune response mounted by different therapies (107), and also the mechanisms by which TNFα generates resistance to immunotherapy, such as monoclonal antibodies against cancer cells or immune checkpoints, and adoptive cell therapy elsewhere (108). With regards to immune exclusion, it is known that TNFα may impair the infiltration of effector T cells by negatively regulating the formation of high endothelial venules (HEVs) (109). These HEVs are in close contact with tumor cells and tertiary lymphoid structures (TLS). It has been proved that tumors with high density of HEVs and TLS have enhanced proportion of effector T cells infiltrating the tumor core. Moreover, this has been proved to correlate with favorable clinical outcomes (110, 111). TNFα has also been described to enhance the production of Fas and its ligand FasL on CD8+ T cells (112, 113), which trigger the apoptosis of neighboring CD8+ T cells (114). Our team has demonstrated that soluble TNFα (sTNFα) secretion by HER2+ breast cancer cells induces resistance to the anti-HER2 therapy trastuzumab, by upregulating the expression of the transmembrane glycoprotein mucin 4 (MUC4) through activation of the NF-kB pathway (31, 115). MUC4 belongs to the membrane-bound family of mucins and has two non-covalently associated subunits encoded by a single gene (116). The extracellular subunit, MUC4α, is hyperglycosylated and it favors metastatic dissemination as it confers antiadhesive properties to the tumor cells. The transmembrane subunit MUC4β, contains two EGF-like domains in the extracellular portion that can interact with HER2, preventing its internalization and therefore enhancing its signaling (117). As trastuzumab binds to the juxtamembrane region of HER2 (118), MUC4 with its heavy glycosylation patterns shields trastuzumab epitope on the HER2 molecule hindering its binding and therapeutic effect (119). MUC4 has been proved to interact with HER2 and even to induce its phosphorylation and activation (120). It also promotes HER2 and HER3 translocation to the cell surface, increasing the number of available receptors and keeping them anchored to the membrane during longer periods of time (121). As a consequence, the signaling cascade of the HER2/HER3 heterodimer through activation of PI3K is enhanced (122), causing an increase in cell proliferation and survival in HER2-positive breast cancer (123). In a previous report, we have also demonstrated that MUC4 has a key role in the immune response against the tumors, since it generates an immunosuppressive TME characterized by an increased infiltration of MDSCs, poor NK cells activation and degranulation and an increased proportion of protumoral M2-like macrophages (31). Also, we demonstrated that blocking sTNFα with a dominant negative molecule, INB03, overcomes trastuzumab resistance and remodels the TME into an immunocompetent one, characterized by a decrease in MDSCs proportion, an increase in NK cell activation and degranulation and an enhanced macrophage infiltration and polarization to the antitumor M1-like subtype (31). Moreover, we have proved that MUC4 is an independent biomarker of poor disease-free survival in HER2+ breast cancer patients treated with adjuvant trastuzumab (115). Our studies also focused on the role of MUC4 regarding TILs, since this glycoprotein is heavily glycosylated. We demonstrated that MUC4 acts as a physical barrier for immune exclusion in TNBC and HER2+ breast cancer, since tumor samples from patients with MUC4+ tumors evidenced scarce or null presence of TILs, while patients with MUC4- tumors evidenced abundant TILs on their TME (31, 124). Moreover, mucins can interact with inhibitory receptors like ICAM-1 on T-cells, causing anergy and impaired antigen recognition, and with siglecs on antigen-presenting cells (125, 126). Overexpression of mucins on tumor cells create steric hindrance for therapies, mask the detection of tumor-associated antigens and prevent lysis of the tumor cells by immune cells, generating immune tolerance (126–129). Also, it has been demonstrated that mucins expressed by tumor cells interact with leukocytes in the TME and facilitate the colonization of disseminated cells, since aberrant glycosylation promotes the expression of ligands for selectins expressed by leukocytes and platelets, normally used for adhesion (130). Therefore, mucins form aggregates with these cells and promote metastasis (121, 131). Particularly, it has been described that MUC4 protects disseminated cells from the immune recognition by hiding with its glycosylations the corresponding immunogenic antigens on tumor cells (121), and that, by physically interacting with platelets and macrophages, it able to enhance survival and extravasation (132).

Moreover, proteins with known barrier functions have been described to promote immune exclusion (103). Examples of these proteins are filaggrin, TACSTD2 and desmosomal proteins like desmocollin 3, dystonin, desmoplakin, periplakin, plakophilin 3 and junction plakoglobin. In healthy tissues, these proteins are usually expressed in the outer layer of the skin and play a crucial role as mechanical barriers that protect our body from pathogens. However, Salerno and collaborators found that a subset of metastatic melanomas and ovarian carcinomas express high levels of genes encoding these proteins. Moreover, they prove that the expression of genes encoding proteins with barrier functions correlated with a lack of T cell infiltrates and immune related signatures in multiple data sets. What is more, overexpression of the barrier molecules was shown to predict worse survival for melanoma and ovarian cancer patients across various clinical subsets (103). Melanoma patients that received adjuvant therapy and did not have overexpression of barrier molecules showed improved survival, which may suggest a potential predictive value for this subset of proteins. Particularly, Chen and collaborators described by means of cellular experiments that filaggrin is an oncogene in bladder urothelial carcinoma (BLCA), and that a knockdown of this barrier protein suppressed BLCA cell proliferation and promoted apoptosis (133). Furthermore, based on TCGA data sets the authors proved that BLCA patients expressing the wild-type isoform of filaggrin presented less overall survival than patients expressing mutated forms of the barrier protein, consistent with the fact that higher tumor mutation burden favors responses to immunotherapy (133). T cell infiltration to the tumor bed was modulated by filaggrin mutational state, since patients with wild-type filaggrin showed significant down-regulation of CD4+ naïve T cells, central memory T cells, and natural killer T cells, while Th1 cells were significantly upregulated. Also, these patients showed poorer response to ICB (133). Another recent study evaluated multiplex immunofluorescence histology of tumor samples from 65 advanced melanoma patients, and proved that the expression of another barrier molecule periplakin negatively correlates with response to pembrolizumab (134). Decreased expression of periplakin and increased proportion of CD103+ CD8+ T cell infiltration correlated with improved survival in these patients, so authors propose periplakin as a predictive biomarker to ICB. Moreover, Pai and collaborators proved that the expression of another barrier molecule, the desmosomal protein dystonin, was inversely correlated with the Th1-like immune signature in patients with metastatic melanoma and ovarian cancer (45), and was associated with worse prognosis in patients with melanoma (103). Particularly, dystonin expression allowed the authors to identify tumors with null expression of the Th1-like immune signature, suggesting no interaction between these immune cells and cancer cells. Interestingly, dystonin expression identified a subset of melanoma patients with no CD8+ gene signatures. However, this was not associated with decreased patient survival, which adds to the idea that the presence of T cells within a given TME is not enough to predict prognosis, and that a better approach could be to study the ratio between lymphoid and myeloid or other cellular infiltrate to better define prognostic significance (135–137). These findings raise the attention to barrier molecules as future potential biomarkers for defining T cell accessibility to the tumor core and, consequently, ICB responses.

#### 3.1.2 Collagen and hyaluronic acid

Another important component of the ECM is collagen, which distributes between the cells as fibers. Historically, collagen was demonstrated to be a passive barrier to resist tumor cell infiltration and establishment. However, the role of collagen in cancer progression, immune escape and poorer patient outcome is now widely studied (138–141). Collagen I, III and IV are the predominant types in forming the scaffolds of the TME (142–144). Upon tumor progression, the ECM remodeling of collagen fibers which can be degraded (145) and later re-depositioned (143, 146), can promote tumor infiltration, angiogenesis, invasion and migration (70, 71, 146, 147). How collagen can be a double-edged sword in tumor progression, both inhibiting and promoting tumor progression at different stages of cancer development has been exhaustively reviewed elsewhere (148). In this section, we will focus on the role of collagen fibers as a contributor to immune exclusion.

A recent article from Kuo-Sheng Hsu and collaborators has proven that the survival of cancer cells depend on the uptake of collagen I into their associated stroma (149). Also, evidence indicates that breast tumors with dense collagen depositions correlate with a worse patient’s outcome (150). Moreover, it has been shown that collagen XIII expression is increased in human breast cancer samples compared to normal mammary gland tissue, and increased levels of collagen XIII mRNA correlate with short distance recurrence free survival (151). One of the mechanisms contributing to collagen I-mediated tumor growth promotion is its ability to mediate immune cell exclusion (152). It has been demonstrated that interaction of collagen fibers and immune cells account for inhibition of their antitumor activities (153), and Salmon et al. revealed that the stromal ECM, particularly fibronectin and collagen fibers, influences antitumor immunity against human lung tumors by controlling the localization and migration of T cells (154). Movement of cells towards and within the ECM is prevented through an unusually dense and stiff composition. Salmon stated that it was the higher density of the ECM in the tumor islets that caused T cells to preferentially migrate to the tumor stroma rather than infiltrate the islets. Other researchers also highlight that the remodeling of the ECM that increases tumor stiffness can mechanically activate pathways that lead to tumor progression and dampen T cell infiltration to the tumor islets (155–158). Particularly, studies have demonstrated that T cells are impaired to infiltrate from the stroma to the tumor core of PDAC when the ECM density is high (159, 160). Moreover, it has been stated that tumor fibrosis (accumulation of collagen type I and III fibers in the tumor ECM) is associated with a variety of malignancies (142, 143, 161) and particularly correlates with worse prognosis in breast cancer (162). Also, tumor fibrosis not only increases metstasis risks in different tumor types (163, 164), but it is also necessary for the successful establishment of metastasis foci (165, 166). When fibrosis is highly extensive, for example in PDAC, the scar-like ECM acts as a physical barrier to cytotoxic T-cell infiltration into tumors (167). Collagen type I is the most abundant type of collagen fiber present in the ECM (168). Researchers proved that loose areas of fibronectin and collagen usually facilitate T cell infiltration, while dense and stiff ECM reduce T cell velocity and migration (167). In line with this, it has been proved that central fibrosis in tumors exclude immune cells from CRC metastases (169). One of the collagen receptors, the discoidin domain receptor 1 (DDR1), has also been studied in this regard. The different roles of DDR1 in cancer promotion are summarized in **Table 1**. This receptor has an intracellular domain with tyrosine kinase activity (170), which is triggered by collagen binding and participates in different downstream signal transduction (170, 171), but has no impact on tumor growth (152). In contrast, researchers have found that it is the extracellular DDR1 collagen-binding domain (ECD), the one required for tumor growth in immunocompetent hosts (152). As it happens with collagen, DDR1 also has paradoxical and context-depending roles in cancer. Some authors have described DDR1 as a tumor suppressor, as it induces apoptosis of basal-like breast cancer cells in 3D collagen I matrices (172–174) and in luminal breast cancer cells in young collagen 1-enriched ECM (175). However, when collagen 1 ages, DDR1 no longer promotes apoptosis and the tumor keeps growing. On the other hand, several publications suggest that DDR1 overexpression at protein levels has shown to be associated with cancer progression (176–179) and metastases (180), and the increase of DDR1 mRNA levels was proved to be associated with worse overall survival in all subtypes of breast cancer patients, and particularly in TNBC (152). Regarding invasion, Juin et al. demonstrated that DDR1 plays a key role in the formation and matrix-degradation ability of F-actin invadosomes, promoted by collagen I fibrils (181). Bravo-Cordero and colleagues proved that the DDR1/STAT1 axis favors dormancy by triggering collagen III expression. However, changes in the alignment of collagen III fibers to a more linear orientation may awake tumor cells from dormancy and reactivate proliferation and metastasis (182). Sun and collaborators demonstrated that DDR1 knockout (KO) tumors were impaired to grow in immunocompetent mice, but were capable of growing in immunodeficient hosts. However, depleting CD8+ T cells allowed DDR1 KO tumors to grow at the same rate as wild-type ones in immunocompetent mice (152). Moreover, the authors proved that DDR1 KO tumors exhibited an increase in CD4+ and CD8+ activated T cells, compared to DDR1 wild-type tumors. What is more, when representative tumor samples from each experimental group were analyzed by multiplex immunofluorescence staining, authors found that CD8+ T cells were largely restricted to the tumor margins of DDR1 wild-type tumors, a phenomenon that was also evident by other researchers in similar mouse models of TNBC (183). Strikingly, DDR1 KO tumors showed an increased infiltration of CD8+ T cells on the tumor core, suggesting that DDR1 is a key factor of the TME that physically restricts T cell infiltration. Adding to this, it has been proved that both mRNA and protein levels of DDR1 negatively correlate with genes that define anti-tumour immunity, a gene expression signature for intratumoral T cell accumulation, CD8+ T cell signature scores, and the cytolytic effector pathway (59, 184). The authors found that in TNBC patient samples which have never received any treatment, the percentage of DDR1 positive cells in DDR1 high tumors inversely correlated with the abundance of infiltrating CD8+ T cells, which were circumscribed to the tumor margin rather than present in the tumor core. In contrast, tumors classified as DDR1 low did not show evident differences between the abundance of CD8+ T cells in the tumor core or at the margins. Moreover, when tumor samples were stratified according to the immune phenotype, all tumor samples classified as immune-excluded were DDR1 high and most tumors classified as non-excluded were DDR1 low, confirming the role of tumoral DDR1 in immune exclusion. Finally, with respect to the role of DDR1 in the ECM, it was proved that DDR1-ECD promotes the alignment of collagen fibers, reinforcing the defenses of tumors against immune infiltration (152).

**Table 1:** Roles of DDR1 in promoting cancer.

Contribution to cancer	Reference
Intracellular domain with tyrosine kinase activity	(157, 158)
Extracellular domain promotes tumor growth	(139)
Promotes cancer progression and metastases	(159–163)
Correlates with worse overall survival in breast cancer patients	(139)
Restricts CD8+ T cells to tumor margins	(139, 164)
Correlates with immune-excluded tumor signatures	(139)
Promotes alignment of collagen fibers in the ECM	(139)
Controls linear invadosome formation and matrix invasion	(168)
Participates in tumor cell dormancy through collagen III-rich ECM	(169)

ECM alignment also plays a key role in controlling immune cell migration (154, 185, 186). Particularly, a collagen-alignment signature could be a prognostic factor for the survival of patients with breast cancer (187). The migratory capacity of T cells was impaired in fresh human tumor explants due to the architecture of collagen fibrils that act both as a guiding path to lead T cells out of the tumor core and as a physical barrier that prevents their entrance, leading to a T cell excluded profile (154, 188). Moreover, it has been recently demonstrated that T cell infiltration inversely correlates with stiffening and high density of ECM in different preclinical murine models of pancreatic, breast, and bile duct carcinomas (189). The authors proved that ECM stiffness measurements correlated with tumor growth and ECM crosslinking of collagen fibers. They showed that interfering with collagen crosslinking into fibers by inhibition of the lysyl oxidase (LOX) enzyme reduces ECM content and tumor stiffness, improving T cell migration and improving the efficacy of anti-PD-1 blockade. Combination of anti-PD-1 and LOX inhibition resulted in an increased accumulation of effector CD8+ T cells in the tumor and significant delays on tumor progression in a pancreatic cancer mouse model (189).

Hyaluronan (HA) is a linear glycosaminoglycan, composed of repeating disaccharide units of D-glucuronic acid and N-acetyl-D-glucosamine (190). HA is a conspicuous component of the ECM, where it possesses several functions both in physiological and pathological conditions such as morphogenesis, tissue repair, inflammation and tumorigenesis (191–194). In healthy tissues, HA presents high molecular weight (HMW-HA) (>1,000 kDa) and has a structural function. HA fragmentation is critical during inflammation, tissue-remodeling processes, and cancer. HA fragments of 200-1000 KDa are called low molecular weight HA (LMW-HA), and fragments <10 kDa are HA oligosaccharides (195). HA levels and molecular size mainly depend on a fine tune between the expression of the enzymes involved in its synthesis (hyaluronan synthases) and its degradation (hyaluronidases) (196). Hyaluronan levels are increased in different solid tumors and associated with tumor aggressiveness and progression, reviewed elsewhere (197, 198). HA forms a hydrogel-like matrix surrounding the tumor cells acting as an exclusion barrier and, thereby, inhibiting the permeation of different drugs (such as chemotherapeutics or monoclonal antibodies) as well as limiting the accessibility of cells from the immune system. A HA-rich matrix confers resistance to anti-HER2 therapy with trastuzumab in breast cancer cells due to HA-induced masking of HER2 and to inhibition of NK cells access to the tumors. Depletion of HA levels by treatment with a pegylated version of hyaluronidase, PEGPH20, resulted in increased NK cell access and enhanced ADCC (199–201). The HA role in the immune exclusion of T cells has been reported in PDAC, which are characterized by HA accumulation. Indeed, depletion of HA by PEGPH20 in combination with a whole-cell PDAC vaccine modulated myeloid cell function by inhibiting the CXCR4 immunosuppressive signaling axis and lead to enhanced T cell infiltration with a rise of intratumoral effector memory T cells (202). Another study demonstrated that treatment with the inhibitor of hyaluronan synthesis 4-methylumbelliferone, promoted infiltration of inoculated γδT cells into tumor tissue and, consequently, suppressed the growth of PDAC (203). In both studies, PDAC stroma remodeling by HA depletion presented an effective immunosensitizer effect. Despite HA function as an exclusion barrier, HA exerts immunomodulatory actions by interacting with receptors including CD44, RHAMM, or TLR4. HA can modulate both the innate and the adaptive immune responses and its final effects depend mainly on the molecular weight of HA, the immune cell type, and the receptor involved. While native HA appears to be anti-inflammatory and anti-tumorigenic, HA fragments and oligomers are pro-inflammatory and pro-tumorigenic (204). The effects of both HMW-HA and HA fragments on TAM function has been extensively reviewed elsewhere (205, 206). While the role of HA on TILs remains to be explored, HA is an important mediator of T cell trafficking. HA/CD44 interaction is known to facilitate the rolling and extravasation of T cells to inflammatory sites (207). Furthermore, HMW-HA, but not LMW-HA, induced the immunosuppressive actions of regulatory T cells. Indeed, HMW-HA enhanced Foxp3 expression and IL-10 production by Tregs (208, 209). Altogether, these reports point out a crucial role of HA in the regulation of T cell recruitment and function in the TME. Further studies aimed to investigate the levels and the molecular weight of HA polymers within the TME will help to elucidate their specific cellular functions *in vivo*.

#### 3.1.3 Aberrant vasculature

Tumor vasculature density and molecular signature has also been shown to have a role in T cell infiltration (210–212). The whole process of T cell migration into target tissues is reviewed elsewhere (213, 214); the last stage of leukocyte infiltration is called transendothelial migration and extravasation. This process usually takes place in HEVs, and it has been reported that tumors with higher amounts of HEVs will promote increased infiltration than tumors with aberrant vasculature formation (210, 211). Tumor-associated endothelial cells play key roles during tumor development such as angiogenesis, regulating vessel permeability, transportation of the immune cells, intravasation and extravasation of tumor cells during dissemination (12). Leukocyte attachment, rolling and transmigration into tissues is regulated by endothelial cells, through the expression of a series of adhesion molecules, for example the intracellular adhesion molecule (ICAM), the vascular cell adhesion molecule (VCAM) as well as E- and P-selectin (12). However, TILs infiltration to the tumor core is affected by a dysregulation of these receptors on the tumor-associated vasculature. Aberrant tumor vasculature is characterized by collapsed blood vessels that are unevenly formed and are often leaky. These vessels usually induce hypoxia, scarce drug trafficking and immune infiltration to the tumor core (215). Also, the endothelium expresses molecules that act as ligands for T cells, which can either inhibit or stimulate immune cell extravasation (216). One example of molecules that stimulate cell extravasation is the extracellular superoxide dismutase 3 (SOD3), an enzyme expressed within the TME that improves the aberrant functions of tumor-associated vasculature by stabilizing hypoxia-inducible factor 2 (HIF2α) that, in turn, induces the expression of vascular endothelial cadherin (212, 217). This signal transduction cascade causes a reduction in vascular leakage and increases blood flow towards the tumor (217). However, SOD3 expression is downregulated in several tumors, including pancreatic, colorectal, lung and breast cancer (218–220). Mira and collaborators have demonstrated that SOD3 enhances effector T cell transmigration and extravasation through the upregulation of WNT ligands, induced by HIF2α. WNT ligands cause the increase of laminin subunit alpha-4 (LAMA4) expression in endothelial cells, a laminin that participates in the maturation of microvessels (221) and gives permissive signals in favor of the transendothelial T cell extravasation (44). Interestingly, SOD3 does not trigger permissive signals for migration of Tregs or myeloid cells. Since SOD3 expression is associated with an increased infiltration of CD8+ T cells and improved outcomes in patients with stage II CRC, the authors claim that this regulatory mechanism of vascular normalization could have clinical implications (217). On the other hand, there are negative signals that inhibit effector T cell adherence to the tumor endothelium and impair T cell recruitment and extravasation to tumor sites. This phenomenon is known as endothelial cell anergy. For example, endothelin B receptor (ETBR) synthesis has been proved to interfere with the adhesion of T cells to the endothelium and, consequently, a successful transendothelial migration and extravasation process. ETBR expression has been proved to be increased in the endothelium of human ovarian cancer and inhibits T cell infiltration to the tumor core. Authors have demonstrated that this inhibition on T cell infiltration can be reversed through treatment with the ETBR neutralizing agent BQ-788 (222). Another overexpressed factor on the tumor-associated vasculature that has been implicated in TILs exclusion is Fas ligand (FasL), produced by murine and human tumors, like ovarian, colon, prostate, breast, bladder, and renal cancer (223). T cells express Fas receptor on their membrane and, upon FasL binding, CD8+ T cells undergo apoptosis. However, this effect has not been observed in Tregs, since they also express c-FLIP on their cell membrane, a protein capable of inhibiting apoptosis. As a consequence, if the tumor-associated vasculature expresses high levels of FasL, although CD8+ T cells may be recruited to the tumor core they are not able to penetrate successfully, and Tregs are preferentially recruited instead (223). Authors also analyzed cohorts of ovarian, colon, bladder, prostate, and renal cancer patients and found that intraepithelial CD3+ or CD8+ cells correlated with significant increases in overall survival, and that high percentages of tumor vessels with FasL+ inversely correlated with intraepithelial CD3+ cells presence. This was further analyzed and authors found that the lack of CD3+ cells was mostly due to a lack in intraepithelial CD8+ TILs, since tumors with FasL+ vessels exhibited abundance of intraepithelial FoxP3+ cells. This data indicates that tumor endothelium induces FasL expression and selectively increases Treg:effector T cell ratio on the tumor core, allowing for CD8+ T cell exclusion in several solid tumor types (223). In line with this, in animal models the inhibition of FasL increased significantly the infiltration of effector T cells and resulted in tumor growth suppression. The authors described that FasL expression is regulated by VEGF, prostaglandin E2 (PGE2), and IL-10. They claim that blocking tumor angiogenesis promotes effector CD8+ T cell infiltration by limiting their tumor vasculature FasL-mediated apoptosis. This is relevant particularly for *ex vivo* activated T cells used for ACT or endogenous activated T cells for cancer vaccines, as activated T cells are prone to undergo apoptosis mediated by FasL. It is known that VEGF and fibroblast growth factor 2 (FGF2) can cause endothelial cell anergy by downregulating the expression of ICAM1 and VCAM1 on the endothelium, two key adhesion molecules for T cell infiltration (224). Moreover, increased expression of some growth factors like VEGF, platelet-derived growth factor (PDGFC) and placental growth factor are able to promote tumor angiogenesis (225, 226). Moreover, it was proved that VEGF can inhibit endothelium activation induced by NF-κB signaling, and impair the T cell infiltration, by blocking the production of chemokines CXCL10 and CXCL11 secreted by tumor and stromal cells (227). These chemokines correlate to T cell abundance in the tumor core of lung (228, 229), colorectal (230) and melanoma (228) patients. Another factor that can influence T cell infiltration into the tumor nest is the integrin-selectin signature expressed in the tumor vasculature, since they are key players in the stages of leukocyte rolling to achieve successful extravasation into target tissues (231). In this regard, researchers have determined the role of the ECM, particularly integrins, in the preferential localization, migration and activation of T cells in lung adenocarcinomas, TNBC and HER2+ breast cancer tumors (154, 232). Particularly, integrin subunit alpha 5 (ITGA5) has a crucial role in promoting cancer cell invasion, metastasis and it was recently proved to be overexpressed in gastrointestinal tumors and was associated with worse overall survival and disease-free survival in colorectal, pancreatic, gastric and liver cancers (233). What is more, expression of ITGA5 was associated with an increased infiltration to the tumor core of CD4 + T cells, macrophages, neutrophils and dendritic cells. Also, ITGA5 expression measured by immunohistochemistry in gastrointestinal tumor samples was proved to be associated with gene expression signatures related to immunotolerant populations, such as TAMs, Th2 cells and protumoral M2-like cells. Authors claim that ITGA5 may have a potential role in polarizing macrophages to the M2 subtype and promoting the activation of tolerogenic lymphocytes (233). ITGA5 is not only expressed in tumor cells, but it has also been reported to be expressed in CAFs (234), TAMs (235) and chimeric antigen-receptor expressing T cells (233). CAFs have a clear role in regulating the recruitment of immune cells and their functions with regards to their anti-tumor responses (167, 236), which will be discussed afterwards in this review. ITGA5 expressed in the surface of TAMs and T cells can directly regulate recruitment and alternative activation of immune cells, and its interaction with other integrins is crucial for the integrin-mediated signaling pathways, leukocyte migration and cell-matrix adhesion (237). This data proves, once more, the importance of the interplay between vasculature and the ECM in immune exclusion and response to therapy.

### 3.2 Immunosuppressive cells

**Figure 2:**
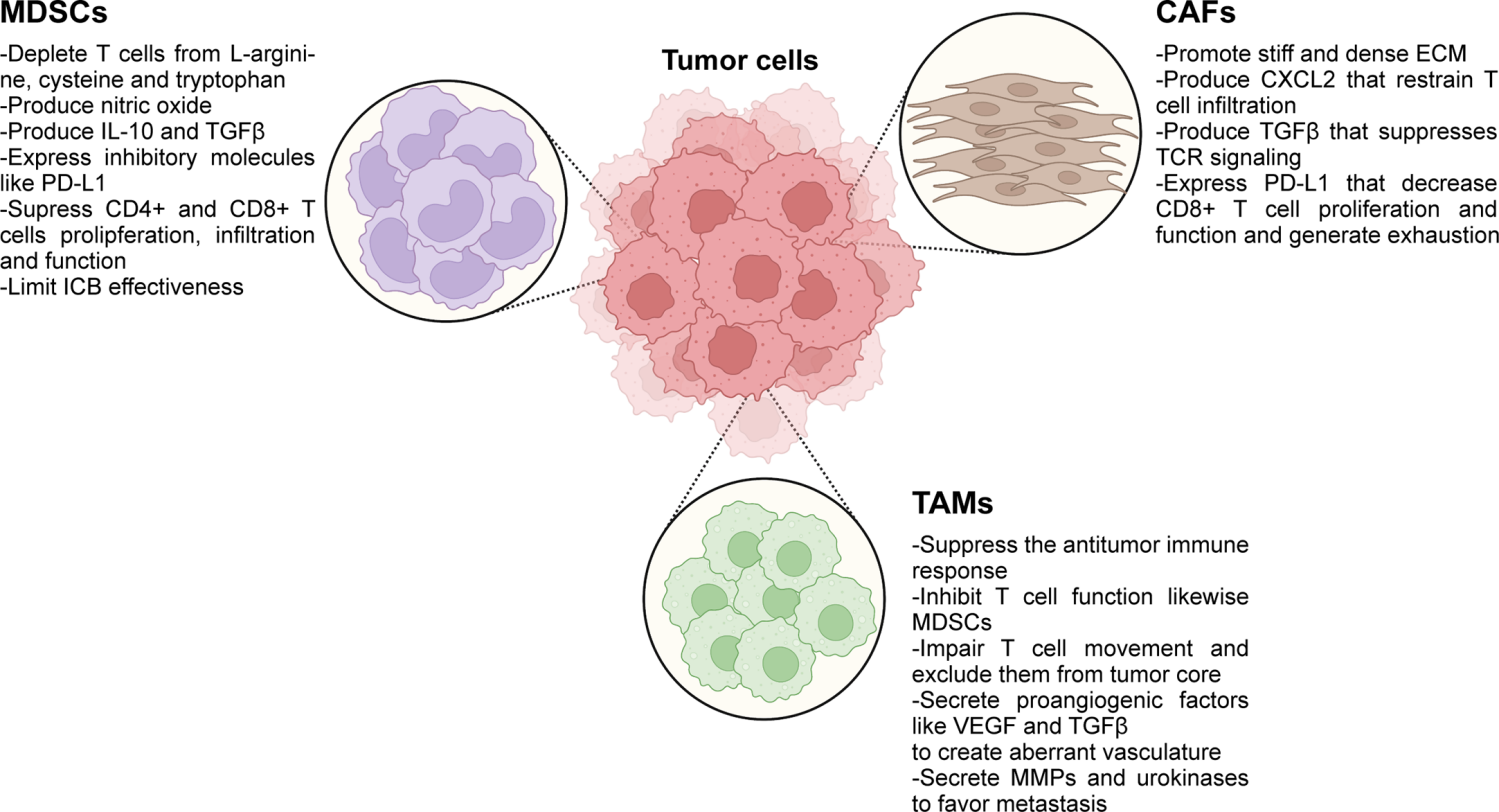
Immunosuppressive cells present in the TME play active roles in immune exclusion and immunosuppression. ICB, immune checkpoint blockade; ECM, extracellular matrix; MDSCs, myeloid-derived suppressor cells; TAMs, tumor-associated macrophages; CAFs, cancer-associated fibroblasts; MMPs, matrix metalloproteinases. Created with BioRender.com.

Numerous preclinical studies have proposed several stromal cell types of the TME, like CAFs, MDSCs and TAMs as the key players for restricting infiltration of T cells in the tumor core (135, 238–243). In this section we discuss the most recent evidence on the mechanisms by which these cells promote immune exclusion and therapy failure. The main roles of immunosuppressive cells in immune exclusion are summarized in **Figure 2**.

#### 3.2.1 Myeloid cell barrier

Thanks to their plasticity, myeloid cells are able to modify their differentiation conditioned by tumor factors to achieve an immunosuppressive phenotype that fosters immune evasion. MDSCs are produced by an abnormal myeloid progenitor development in the bone marrow induced by high levels of factors produced by tumors and their stroma, such as G-CSF, GM-CSF, IL-6, and VEGF, which activate Stat3 and Stat5 favoring their differentiation to the detriment of normal monocytes, macrophages and dendritic cells (244–247). The relationship between cytokines and immunosuppressive cells was clearly depicted in pancreatic cancer and liver metastasis, as tumor-GM-CSF induces the activation and expansion of MDSCs, which in turn, suppress T cell function (242, 248). MDSCs enter the circulation from the bone marrow and infiltrate the primary or metastatic tumor site in response to gradients of chemokines such as IL-8, CCL2, and CXCL12 (249–251). Once in the TME, different factors such as hypoxia, adenosine, the activation of the endoplasmic reticulum stress response pathway, among others, increase the immunosuppressive activities of MDSCs (252–254). Two major subpopulations have been described in humans and mice: the polymorphonuclear MDSC (PMN-MDSC) and the monocytic MDSC (M-MDSC). The former is derived from granulocytic precursors and are similar to neutrophils, while the latter are derived from monocytic precursors and are similar to monocytes. In this complex scenario, Long et al. described that erythroid progenitor cells can switch to the myeloid lineage and differentiate to erythroid-differentiated myeloid cells (EDMC), which suppress T cell function and promote anemia (255). EDMC in the TME together with anemia, are described as biomarkers of poor response to ICB therapy in several cancer types (255). Recently, immunotherapy has burst in the oncology arena as a promising approach to exploit the immune system to combat cancer, focusing mainly on the cytotoxic capacity of CD8+T cells. On the one hand, the ICB treatment is prone to induce an active immune response, reinvigorating *in vivo* exhausted endogenous T cells. On the other hand, other therapies rely on a passive strategy by transferring autologous T cells that were genetically engineered to express chimeric antigen receptors. MDSCs have been recognized as a barrier that limits ICB effectiveness (256, 257). MDSCs manage to suppress CD8+ and CD4+ T cell proliferation and function by depleting nutrients, such as L-arginin, cysteine and tryptophan, by inducing the production of nitric oxide (NO) by the inducible nitrous oxide synthase (iNOS), the production of the immunosuppressive cytokines IL-10 and TGF-β, and by expressing the inhibitor molecule PD-L1 (258–262). In addition, in a mouse melanoma model it was demonstrated that MDSCs impair the infiltration of activated CD8+ T cells (240). In a meta-analysis, elevated numbers of MDSCs in the circulation were found to be an independent indicator of poor outcome in patients with solid tumors (263). In melanoma patients subjected to ICB treatment, a basal higher level of PMN-MDSC was proved to be present in progressive disease vs. patients that evidence clinical benefit. The patients that responded to therapy exhibited an increase in circulating MDSCs after ICB treatment, suggesting that MDSCs count could be considered as a prognostic biomarker of ICB effectiveness (264).

The other myeloid cells in the TME scenario are macrophages. Macrophages are present in every tissue to maintain their homeostasis. The tissue-resident macrophages originate from monocytes from the bone marrow but are also derived from precursors that seed the tissues upon different waves of embryonic hematopoiesis and are locally proliferating (265). In particular, a glioblastoma can have 30% of its tumor mass composed of bone marrow–derived macrophages and its abundance is related to tumor grade (266). In a scenario of acute or chronic inflammation (this latter case, includes cancer) bone marrow-derived monocytes migrate to the inflamed tissue attracted by CCL2 and CSF 1, and differentiate to macrophages (267). The macrophages present in tumors are defined as tumor-associated macrophages (TAMs) and can also derive from M-MDSCs as precursor cells (268). Indeed, the cancer-related inflammation is promoted by TNFα secreted by TAMs that induces constitutive activation of NF-κB in tumor cells, which promotes their survival and invasion (269). The ability of the TME to polarize the recruited monocytes to TAMs instead of to dendritic cells has been recently uncovered and is due to the production of retinoic acid by tumor cells (270). Macrophages are plastic and signals from the TME, derived from tumor cells, lymphocytes and stroma cells, condition their fate to acquire either immunosuppressive or immunostimulant function. The “classically activated”, M1-like or antitumoral macrophages are originated by interferon (IFN)-γ or microbial stimuli and produce IL-12, which polarize CD4+T cells to a type I response. In addition, they have cytotoxic activity against microorganisms and tumor cells. On the other hand, the “alternatively activated” macrophages, M2-like or protumoral macrophages differentiate after IL-4/IL-13/IL-10/TGFβ stimuli, have high IL-10 production, downregulate M1-like macrophages´ function and are associated with repair of wound tissues (271, 272). Macrophage polarization conditioned by TME has been recently reviewed (273). It is generally accepted that TAMs have an M2-like phenotype that can promote cancer initiation, progression and favor metastasis while suppressing the antitumor immune response (274–276). In particular, TAMs inhibit CD8+ T cell activation and function in a similar way to that was described for MDSCs: TAMs promote L-arginine depletion in the TME, secrete immunosuppressive cytokines, including IL-10 and TGF-β, and express the inhibitory molecules PD-L1, PD-L2, CD80 and CD86. This profile hijacks the anti-PD-1/PD-L1 therapies, as has thoroughly been reviewed elsewhere (277). TAMs also act as a trap for CD8+T cells. In human lung squamous-cell carcinoma it has been demonstrated that the exclusion of CD8+T cells from the tumor nest was not only due to the density of the ECM but also induced by a long-lasting interaction between TAMs and CD8 T cells which impairs T cell movement (278). In addition, TAMs secrete C-chemokine ligand (CCL)-2, CCL-3, and other members of the CCL family that promote the recruitment of regulatory T cells (Treg) and type 2 conventional dendritic cell (cDC2) HLA-DR^low^ which, upon their interaction in hypoxic conditions, results in a loss of antigen-presenting capacity of cDC2 (279). TAMs also secrete angiogenic cytokines such as VEGF-A, TGF-α, TGF-β, EGF, and PDGF that allow aberrant tumor-associated vasculature (274), which has been described to play a key role in immune exclusion, as reviewed above. Metastasis is a multistep process that allows tumor cells to survive and proliferate in distant organs. In this respect, TAMs secrete matrix metalloproteinases and urokinase that remodel the TME allowing local invasion of cancer cells that form adherens junctions to the ECM. Moreover, the M2-like macrophages are an important component of the tumor microenviromment of metastasis (TMEM) doorways. This structure is composed of perivascular M2-like macrophages, endothelial cells and tumor cells expressing Mammalian enabled actin-regulatory protein (MENA). The tumor cell interaction with M2-like macrophages flow through the tumor cells *via* the TMEM doorway releasing them to the bloodstream to accomplish another step of the metastatic cascade (280–282). These niches, where the dissemination trajectories of tumor cells occur, are immune desert and enable the successful migration of tumor cells to disseminate through the host (283). Another layer of complexity of TAM impact on the TME relies on their ability to remodel tissue by interacting with cancer-associated fibroblasts (CAFs) promoting collagen production and fibrosis, fostering the exclusion of T cells and causing failure of ICB (284). As CAFs secrete high levels of IL-6, it induces Stat3 activation and macrophage polarization to M2-like increasing the immunosuppressive tumor milieu (285).

#### 3.2.2 CAFs and the ECM

The major producer of ECM in solid tumors are the CAFs (286), which are mainly co-opted resident (287) or recruited fibroblast-like cells that undergo reprogramming by the tumor (288). In several tumors, an increased proportion of CAFs on the TME has been associated with poor prognosis (289), but lately a wider and more specific classification of CAFs subpopulations highlights the idea that not all CAFs are biomarkers of poor outcome (290, 291), and that different CAF subtypes may have different contributions to cancer progression. This is due to the heterogeneity of CAFs, as they can be immunosuppressive or immunostimulatory for T cells. As PDAC is characterized to be surrounded by abundant desmoplasia, it has been crucial to study CAFs subpopulations. Öhlund et al. revealed that pancreatic stellate cells are a main source of CAFs, since they can either differentiate to inflammatory CAFs (iCAFs) or myofibroblastic CAF (myoCAF). These subpopulations were defined based on their protein expression: iCAFs secrete IL-6 and pro-inflammatory factors, while myCAFs have elevated expression of α-smooth muscle actin (αSMA). By RNAseq, authors identified that the proinflammatory genes as *Il6, Il11*, and *Lif*, and chemokines, such as *Cxcl1* and *Cxcl2* were upregulated in the iCAFs, while a TGFβ-response genes were characteristic of myCAFs. In addition, a spatial separation between iCAFs and myCAFs was reported: myCAFs are in contact with tumor cells, while iCAFs are situated far from the neoplastic nest (290). These two subpopulations were confirmed by single-cell RNA sequencing in human PDAC (292). Nevertheless, another subtype of CAFs emerged from scRNA in human and mouse PDAC, the so-called antigen-presenting CAFs (apCAFs). These cells have high levels of major histocompatibility complex (MHC) class II family members and, in a mouse model, showed capacity to activate CD4+T cells (291).

CAFs have shown to have different strategies to restrain T cells to the stroma and prevent them from penetrating the tumor core. One of these strategies is their contribution to the production of a stiff and dense ECM, since it has been demonstrated by live imaging of lung tumor tissue slices that T cells move actively in regions of loose fibronectin and collagen fibers, while dense ECM surrounding the tumor core presented a clear impairment to T cell mobility (154). Interestingly, when collagenase was used to reduce the rigidity of the ECM around tumor islets, an enhanced movement of T cells was observed from the stroma and into the tumor core, in close contact with tumor cells. Another strategy by which CAFs can control T cell infiltration is by means of the production of the cytokine CXCL12. In a preclinical mouse model of PDAC, it was demonstrated that CAFs that express the fibroblast activation protein (FAP) within the ECM produce and secrete CXCL12, which in turn binds to the PDAC tumor cells, coating them (241). This phenomenon has also been reported for colorectal and ovarian cancer (293, 294). Feig and collaborators found that T cells were absent from tumor regions containing cancer cells, which were coated with CXCL12. Moreover, they proved that the main source of CXCL12 production in the TME were the FAP+ CAFs. The administration of AMD3100, an inhibitor of the CXCL12 receptor (CXCR4), highly increased T cell infiltration among cancer cells. What is more, authors claim that AMD3100 synergized with anti-PD-L1 therapy to diminish the proportion of cancer cells within the lesion and to arrest tumor growth (241). CD8+T cells functionality is hijacked by CAFs at different levels. TCR signaling can be suppress by secretion of TGFb produced by CAFs (295). CAFs also express PD-L1 and PD-L1 that decrease CD8+ T cell proliferation, function and probably contributes to T cell exhaustion (296, 297).

## 4 Clinical impact

Given the importance of the TME in treatment response, in particular immunotherapies, in this section we will address the current state-of-the-art of targeting the above-mentioned physical barriers as novel treatments in combination with standard of care therapies and their clinical implications.

### 4.1 Targeting MDSCs

MDSCs are a subpopulation of immature myeloid cells that expand massively during tumor progression and invasion and have immunosuppressive effects and promote tumor immune escape. There are several reports of MDSCs involvement in chemo-, radio- and immunotherapy (257, 298–303), which poses this cell population as an attractive therapeutic target. Numerous strategies are being tested in clinical trials: promoting maturation, differentiation or depletion of MDSCs, prevention of expansion and recruitment, inhibition of their function or their metabolism (299, 304–306), among others.

#### 4.1.1 Maturation, differentiation or depletion

Vitamin D3 showed to stimulate MDSCs maturation and improved the antitumor immune response in patients with head and neck squamous cell carcinoma extending their progression-free survival (307, 308). All trans retinoic acid in combination with immunotherapy was proven to induce MDSCs differentiation into macrophages, granulocytes, and dendritic cells (309, 310) which not only abolishes the immunosuppressive effects but also can reduce the MDSCs number from the blood (311, 312). In respect to MDSCs depletion, chemotherapeutic agents can diminish their number, but it is not specific for this population (313). Therefore, a monoclonal antibody against CD33 conjugated with a toxin was tested in acute myeloid leukemia for depletion of MDSCs in a phase II clinical trial (314).

#### 4.1.2 Prevention of expansion and recruitment

Stat3 activation is crucial for MDSCs expansion and function and its inhibition with an antisense oligonucleotide called AZD9150 reduced MDSCs circulation in peripheral blood of patients with diffuse large B-cell lymphoma (315), and is currently being tested in combination with ICB in solid tumors (NCT02499328). MDSCs are regulated by the same colony stimulating factors (CSF) that modulate normal myelopoiesis (316). It has been demonstrated in preclinical models for different cancers that blockade of GM-CSF/G-CSF can inhibit the accumulation of MDSCs and exert an antitumor immune response while restraining polarization of the macrophages to the M2-like phenotype (317–320). Moreover, combination of GM-CSF/G-CSF blocking agents with ICB and chemotherapy have given promising results (321) which led to clinical trials that test these combinations (322, 323) in prostate (NCT02961257, NCT01499043), melanoma (NCT02071940, NCT02975700), glioblastoma (NCT01499043) and breast (NCT02265536) among others. Naturally, targeting the complex cytokine and chemokine network or their receptors which modulate MDSCs is expected to have great clinical implications. For example, propagermanium (a CCL2 inhibitor) was shown to be safely tolerated by patients with primary breast cancer and an antimetastatic effect was observed (324). On the other hand, a monoclonal antibody against CCL2 showed no antitumor effect as a single agent in patients with metastatic castration-resistant prostate cancer (325). A small molecule inhibitor of CCR2 combined with FOLFIRINOX, the standard of care for patients with PDAC, had promising results, decreasing TAMs and migration of MDSCs (326, 327). In spite of these results, most of the approaches targeting the CCL2/CCR2 axis have disappointing results, and this could be due to the impediment for long term blocking (328). Targeting CXCR1/2 in combination with chemotherapies showed a potent antitumor response, and enhances the efficacy of chemotherapy in an animal (329) model of gastric cancer, and furthermore have shown to improve ICBs therapy in head and neck tumors and (330)oral and lung carcinoma (331, 332),. Numerous CXCR1/2 inhibitors have been tested in clinical trials for cancer patients and are extensively described elsewhere (330). Another key player in MDSCs recruitment and mobilization is VEGF, which is also produced by them to promote metastasis and angiogenesis (333). VEGF expression in ovarian cancer correlated with a poorer prognosis and MDSCs infiltration (334). Different studies have approached the use of anti-VEGF/VEGFR therapies in patients with NSCLC (335), glioblastoma (336) and colorectal (337) cancer and presented good outcomes. Sunitinib, a tyrosine kinase inhibitor that targets PDGFRs and VEGFRs, has shown to inhibit MDSCs immunosuppressive effect in patients with various cancer types (338). Another study in renal cancer patients showed that sunitinib decreases circulating MDSCs (339) by inhibiting VEGF and STAT3 activation (338). To achieve effectiveness, combination treatments that target several of these factors are being tested in preclinical models, for example in a preclinical breast cancer model all trans retinoic acid administration together with VEGFR2 inhibitors and chemotherapy have been tested and not only they diminished MDSCs infiltration, but also exhibited hindered tumor growth (340).

#### 4.1.3 Inhibition of function

These treatments aim to inhibit pathways related with MDSCs immunosuppressive mechanisms. Inhibition of STAT3 through different strategies in preclinical models and clinical trials (316, 341, 342) have been tested in solid tumors with limited effectiveness and unwarranted toxicities (343). The COX2/PGE2 axis is another interesting target since it has been shown to be involved in tumor promotion and invasion in ovarian cancer (344), tumor evasion in colorectal adenoma (345), among others (346). Inhibition of said signaling pathway showed to improve immune response and decreased arginase expression (347, 348) and exhibited great inhibitory effect over the MDSCs population in several cancers (349–351). Combination with immunotherapy has been already reviewed elsewhere (352). Inhibitors of the histone deacetylase have also shown to reduce COX2, arginase and iNOS levels, resulting in an inhibition of the immunosuppressive function of MDSCs. These drugs have been tested in preclinical models (352, 353) and in clinical trials resulting in sustained survival, retarded tumor growth and a positive immunostimulatory effect over CD8+ T cells (354, 355). Phosphodiesterase inhibitors can also alter MDSCs function downregulating iNOS and arginase stimulating antitumor immunity in mice (356) and in patients with HNSCC (357) and melanoma (358).

### 4.2 Targeting TAMs

TAMs account for 30-50% of the TME (359) and promote tumor progression (360, 361) and an immunosuppressive TME depending on their polarization (362, 363). These characteristics make them an appealing target to modulate the TME and obtain tumor regression or increase efficacy of current therapies. Depletion of TAMs, stimulation of their phagocytosis, reprogramming and inhibition of their recruitment are some of the proposed targeted therapies.

Paralleling CAR-T cells, recently, chimeric antigen receptor macrophages (CAR-M) have been developed. CAR-M have shown to polarize macrophages to the M1 antitumoral subtype and to enhance the phagocytosis of cancer cells (364–367), and are also capable of transforming the TME into a pro-inflammatory one, which promotes antitumoral effects in preclinical models (366). CD47 (don’t eat me signal) is a crucial molecule expressed by tumors that impedes macrophage recognition of the tumoral cells, which results in a decreased phagocytosis and in tumor evasion (368). Several works have studied potential therapies directed against CD47 and have shown favorable results (369–372). Regarding TAMs depletion, bisphosphonates have been shown to be effective and they also inhibit M2-like TAMs proliferation and migration, but they do not achieve a durable response (373–377).

Another interesting strategy is TAMs reprogramming. Macrophages can shift between M1-like antitumoral or M2-like pro-tumoral phenotypes, depending on the cytokines present in the microenvironment (378). As it was described for MDSCs, recruitment of M1-like macrophages to the tumor core is an interesting therapeutic idea and given both cells belong to the myeloid population, some of the therapies used for MDSCs also affect TAMs. In line with this, TLR agonists are currently being tested in the clinical setting to repolarize M2-like TAMs to the M1-like subtype (379–384). Moreover, CSF-1/CSFR-1 blockade inhibits TAMs recruitment to the tumor bed and has antitumoral effects in mice and in patients (385–388). The CCL2/CCR2 axis is also important for TAMs recruitment and therapies that target these proteins have shown to be effective in this sense and they also reduce tumor growth (326, 389, 390). Inhibitors of CSF-1/CSFR-1 not only affect TAMs recruitment but also have a reprogramming effect (322, 385, 391, 392). Given the rise of immunotherapies, several strategies that target TAMs are now being tested in combination with ICBs and have shown to improve their effectiveness (375, 393–399).

### 4.3 Targeting CAFs

Lately, CAFs have emerged as an important factor in the regulation of the TME and immunotherapy response. Fibroblast activating protein (FAP) is one of the main targets for depleting CAFs (400), and inhibition of said protein showed good outcomes in mice models (401, 402). However, a monoclonal antibody targeting FAP did not show the expected promising results in a phase II clinical trial of patients with metastatic colorectal cancer (403). Bispecific antibodies of FAP and IL-2 receptor (RO6874281) or 1BBL (RG7826) are being tested in combination with ICBs on clinical trials and have shown to be safe, and preclinical models encourage the advancement due to promising results such as tumor regression, accumulation of CD8+ T and NK cells. Other approaches aim to inhibit pathways related to CAFs activation, for example, focal adhesion kinase (FAK) inhibitors combined with anti-PD-1 had a good effect in preclinical and clinical models (159, 404) (NCT03727880, NCT02758587, NCT02546531). In this sense, inhibition of the platelet derived growth factor (PDGF) (405) and FGFR (406) pathways have shown positive outcomes for the patients but the adverse effects and resistant events remain to be elucidated. On the other hand, depletion of α-SMA+ myCAF induces reduced survival of animals with PDAC and scarce myCAF in human tumor samples was correlated with poor overall survival (407). Therefore, a deep insight of CAFs subtype and their relationship with tumor types should be unraveled before their approval in patients. Recent review highlights the challenges that targeting CAFs has to overcome to reach clinical practice (408, 409).

Some downstream effectors of CAFs have been studied as potential therapeutic targets such as CXCR4/CXCL-12 (410) (NCT02907099, NCT02826486) or TGF-β (36, 97, 411–413). Interestingly, some of them are also being proposed as targets of other cell populations as mentioned above, meaning these therapies affect multiple cell types. Given the recent description and implications of CAFs subsets in the TME remodeling and immunotherapy response, future studies should disclose the role of said subsets and new therapeutic targets will arise.

### 4.4 Targeting collagen

Collagen is a central element of the ECM, and therefore for the TME and immune (414) exclusion, has implications in tumor progression, metastasis, and conditions response to treatment. However, collagen has also shown to exhibit antitumor activity (415). Collagen-binding domains (CBD) are present in collagen and mediate interactions with other proteins (416). Given this, CBD-engineered biomolecules could deliver drugs or monoclonal antibodies to the tumor site and their effect is limited to the ECM, augmenting the therapeutic outcomes and decreasing off-target effects and toxicity (417). Among these examples, CBD fused with the EGFR binding portion of cetuximab showed antitumor activity *in vitro* and longer retention times and enrichment in tumor human epidermoid squamous carcinoma xenografts (418, 419). In the same manner, CBD carrying IL-2 or IL-12 in combination with ICB treatment have been tested, exhibiting promising results in breast cancer (420) and melanoma (421). In another approach, collagen-binding albumin, a protein that accumulates in the TME and is used as an energy source for cancer growth, was conjugated to chemotherapy in combination with ICB in a colon carcinoma preclinical model (422). The treatment not only caused accumulation of the drug on the TME but also generated complete tumor inhibition in breast and colorectal cancer models (423). Recently, a fusion protein of CBD and the Fc of SIRPα (signal regulatory protein α) (423), which targets the SIRPα-CD47 axis, showed accumulation in the tumor tissue, more antitumor activity and an increase infiltration of M1-like macrophages than SIRPα Fc alone, in a xenograft model of NSCLC (424). Collagenase treatment loosens the ECM, allows an improved drug penetration and delivery and provides an antitumor effect (425–427). Alternatives to achieve collagen degradation include activators of matrix metalloproteinases (428, 429), engineered bacteria (430) and armed oncolytic virus expressing ECM components (431–434).

Another strategy is not to deplete collagen, but to modulate its production by interfering with collagen crosslinking. The LOX family of enzymes is critical in collagen crosslinking and therefore ECM stiffness. In fact, it has been shown that aberrant LOX expression is implicated in tumor progression (435) and therapy resistance (436). Therapies targeting LOX have been developed and tested in preclinical models in various cancers showing good results (437, 438). Inhibition of collagen synthesis by antifibrotic drugs loosens ECM architecture allowing greater immune cell infiltration and drug delivery in PDAC (439, 440), NSCLC (441), melanoma (442), among others. There are several clinical trials testing antifibrotic drugs that could potentially be used in cancer treatment in the future (443). Clinical trials testing therapies targeted collagen have been reviewed elsewhere (138).

Recently, it was demonstrated that an antibody against the collagen receptor discoidin (DDR1) disrupts collagen alignment and inhibits tumor growth in TNBC (152). Finally, given that CAFs are the main producers of collagen, therapies targeting this cell population can achieve the above-mentioned collagen-related effects.

### 4.5 Targeting HA

As mentioned above, HA can act as a physical barrier causing both the exclusion of immune cells and of drugs (such as chemotherapeutics and monoclonal antibodies), thus leading to failure of treatment. Finding efficient tools to remodel the HA matrix within tumors is complex and constitutes a matter of intense research nowadays. One strategy proposes to target the hyaluronidase activity with O-sulfated HA, and showed a pronounced effect over invasion in PDAC (444) and prostate cancer (445).

The application of hyaluronidases to degrade HA has also been investigated as a possible anticancer drug alone or in combination with other drugs. Indeed, intravenous hyaluronidase treatment of mice implanted with human breast cancer cells significantly reduced tumor growth (446). Intravenous trastuzumab constitutes the main standard of care for HER2-positive BC since 1998. As an alternative, a subcutaneous trastuzumab formulation was developed containing the recombinant human hyaluronidase, which degrades interstitial HA, enables the administration of large drug volumes subcutaneously, and favors trastuzumab delivery to the circulation (447, 448). After a phase III trial confirmed the comparable efficacy and safety of subcutaneous and intravenous trastuzumab (449), subcutaneous trastuzumab was approved for HER2-positive early BC. This trastuzumab formulation presents several advantages compared with the intravenous trastuzumab including shorter administration times and increased convenience and preference for patients (450). Preclinical studies in pancreatic cancer models demonstrated that the hyaluronidase PEGPH20, besides increasing CD8+ T cell infiltration as stated before, increases chemotherapy effectiveness (451). Based on this, PEGPH20 treatment was assessed in clinical trials for PDAC, where it showed an increase in perfusion and chemotherapy delivery (452). Although early phase studies showed promising results, further clinical studies demonstrated that addition of PEGPH20 to chemotherapy either increased toxicity or failed to achieve an improvement in the progression-free survival or overall survival, so the studies were discontinued for PDAC (452–454). It is worth noting that although HA may be a physical barrier hampering drug delivery, it may also have signaling properties. Therefore, hyaluronidases may also generate HA fragments, which could have pro-tumorigenic (pro-angiogenic) actions and may explain the failure of PEGPH20 treatment in PDAC. Blocking peptides for LMW HA is another interesting strategy to prevent HA to bind its receptors and downregulate signaling, but to date there is not extensive work approaching this task. However, it has been demonstrated that HA-binding peptides can inhibit survival and invasion of breast and prostate cancer cell lines *in vitro* and *in vivo (* 455–457).

Another therapeutic strategy proposes to target HA synthesis by treatment with 4-metylumbilliferone (4-MU), a natural compound that inhibits the expression of HA synthases (152, 458) and therefore downregulates HA signaling. Preclinical studies have shown that 4-MU has antitumoral effects affecting progression, migration, and invasion in several cancers, such as colorectal, pancreatic, prostate, ovarian, breast, melanoma, hepatocellular cancers (459). 4-MU also has shown to reduce metastasis in breast (460), ovarian (461) and skin (462) cancers. Since 4-MU is already approved in Europe for biliary spasms treatment, and given that it decreases HA levels in human participants (463), 4-MU constitutes a promising anticancer agent. Recently, another inhibitor of HA synthesis, the thymidine analog 5′-Deoxy-5′-(1,3-Diphenyl-2-Imidazolidinyl)-Thymidine (DDIT), demonstrated more potent anti-tumorigenic properties than 4-MU in breast cancer (464), further supporting targeting HA synthesis as an antitumoral therapeutic strategy. Further studies investigating the impact of HA-targeting strategies on HA levels, HA molecular weight, and the activation of HA receptors will help to improve current and develop new therapeutic tools aiming HA to tailor HA metabolism in cancer.

### 4.6 Targeting MUC4

MUC4 expression was reported to be higher in various cancer types such as breast, pancreatic, lung, ovarian, bladder and cervical cancer (465). Our group reported that TNFα-induced MUC4 is involved in HER2-targeted therapy resistance *in vivo* and *in vitro*, and that it is an independent predictor of trastuzumab response in HER2-positive breast cancer patients (115). We have disclosed that a dominant-negative protein (INB03) that selectively blocks sTNFα downregulates MUC4 and its administration with trastuzumab turns the immunosuppressive TME into a immunostimulatory one through polarization to M1-like macrophages and favoring the crosstalk between macrophages and NK cells in preclinical models (466). Furthermore, MUC4-positive breast tumors are associated with an immunologically cold phenotype, suggesting that INB03 could be used in combination with trastuzumab and ICBs in these tumors. MUC4 has also been proved to be a biomarker for pancreatic cancer and drives cell tumor proliferation and invasion in PDAC (467, 468). In ovarian cancer MUC4 downregulation potentiates the antitumoral effect of auranofin (469). Moreover, MUC4 expression has been linked to the metastatic potential of the cancer cells (470). Given the aforementioned information and the lack of therapies targeting MUC4, we proposed that TNFα-blocking agents or MUC4-targeted therapies in combination with immunotherapy and the standard of care for each cancer type should be further investigated in the clinical setting.

## 5 Closing remarks

It is clear that the phenomenon of immune exclusion has strong clinical relevance for different tumor types, but has not yet been fully elucidated. We envision that understanding the immune-excluding mechanisms of action of the different components of the TME will open up new avenues of therapeutic approaches. However, a full comprehension of the TME of each tumor type, and even in each patient should be taken into consideration in the clinical setting. In the near future, diagnosis would be performed by promising non-invasive techniques that will help to characterize the TME and select a proper treatment. Moreover, the plasticity of myeloid cells, the depletion of suppressive cells and the reduction of the TME stiffness are different strategies that are now being explored to reshape the immunosuppressive tumor milieu into an immunocompetent one that will render the success of the present IBC.

## Author contributions

SB and RS contributed to the conception of the manuscript. SB, MFM, FLM, RIRC and RS wrote sections of the manuscript. All authors contributed to manuscript revision, read, and approved the submitted version.
